# Ursolic Acid Increases Skeletal Muscle and Brown Fat and Decreases Diet-Induced Obesity, Glucose Intolerance and Fatty Liver Disease

**DOI:** 10.1371/journal.pone.0039332

**Published:** 2012-06-20

**Authors:** Steven D. Kunkel, Christopher J. Elmore, Kale S. Bongers, Scott M. Ebert, Daniel K. Fox, Michael C. Dyle, Steven A. Bullard, Christopher M. Adams

**Affiliations:** 1 Departments of Internal Medicine and Molecular Physiology and Biophysics, and Fraternal Order of Eagles Diabetes Research Center, Roy J. and Lucille A. Carver College of Medicine, The University of Iowa, Iowa City, Iowa, United States of America; 2 Iowa City Veterans Affairs Medical Center, Iowa City, Iowa, United States of America; Wageningen University, The Netherlands

## Abstract

Skeletal muscle Akt activity stimulates muscle growth and imparts resistance to obesity, glucose intolerance and fatty liver disease. We recently found that ursolic acid increases skeletal muscle Akt activity and stimulates muscle growth in non-obese mice. Here, we tested the hypothesis that ursolic acid might increase skeletal muscle Akt activity in a mouse model of diet-induced obesity. We studied mice that consumed a high fat diet lacking or containing ursolic acid. In skeletal muscle, ursolic acid increased Akt activity, as well as downstream mRNAs that promote glucose utilization (hexokinase-II), blood vessel recruitment (Vegfa) and autocrine/paracrine IGF-I signaling (Igf1). As a result, ursolic acid increased skeletal muscle mass, fast and slow muscle fiber size, grip strength and exercise capacity. Interestingly, ursolic acid also increased brown fat, a tissue that shares developmental origins with skeletal muscle. Consistent with increased skeletal muscle and brown fat, ursolic acid increased energy expenditure, leading to reduced obesity, improved glucose tolerance and decreased hepatic steatosis. These data support a model in which ursolic acid reduces obesity, glucose intolerance and fatty liver disease by increasing skeletal muscle and brown fat, and suggest ursolic acid as a potential therapeutic approach for obesity and obesity-related illness.

## Introduction

Ursolic acid is a lipophilic pentacyclic triterpenoid that contributes to the waxy coats on apples, other fruits, and many herbs, including some folkloric herbal medicines for diabetes [1]–[4]. We recently identified ursolic acid in a screen for small molecule inhibitors of skeletal muscle atrophy [5]. In that study, we determined the effects of fasting and spinal cord injury on skeletal muscle mRNA levels in humans, and used that information to generate unbiased mRNA expression signatures of human skeletal muscle atrophy. We then used these signatures to query the Connectivity Map [6] for compounds whose expression signatures negatively correlated with the signatures of human muscle atrophy. Out of >1300 compounds in the Connectivity Map, ursolic acid emerged as the most likely inhibitor of muscle atrophy.

To test the hypothesis that ursolic acid might inhibit muscle atrophy, we studied mice that had been fasted or undergone surgical muscle denervation, and found that ursolic acid reduced muscle atrophy [5]. We then investigated ursolic acid's effect in the absence of an atrophy stimulus by adding ursolic acid to standard mouse chow for 5 weeks. In that setting, ursolic acid induced skeletal muscle hypertrophy [5]. Since the protein kinase Akt (also known as PKB) inhibits muscle atrophy and promotes muscle hypertrophy [7]–[13], we examined ursolic acid's effect on Akt. We found that ursolic acid increased Akt activity in mouse skeletal muscle and in cultured C2C12 skeletal myotubes [5]. In myotubes, ursolic acid increased Akt activity at least in part by enhancing ligand-dependent activation of the insulin receptor and insulin-like growth factor I (IGF-I) receptor.

In addition to causing muscle hypertrophy, genetic interventions that activate Akt specifically in skeletal muscle also increase energy expenditure, reduce adiposity and blood glucose, and impart resistance to diet-induced obesity, glucose intolerance and fatty liver disease [8], [9]. Similarly, we found that ursolic acid reduced adiposity and blood glucose in non-obese mice [5], and others found that ursolic acid reduces total body weight, white fat, glucose intolerance and hepatic steatosis in high fat-fed mice [14], [15]. Based on these considerations, we hypothesized that ursolic acid might increase skeletal muscle Akt activity in a mouse model of diet-induced obesity, leading to muscle hypertrophy, increased energy expenditure and thus, reduced obesity, glucose intolerance and fatty liver disease. In the current study, we tested this hypothesis, and found that ursolic acid increases not only skeletal muscle, but also another tissue that opposes diet-induced obesity, brown fat.

## Results

### Ursolic acid increases skeletal muscle Akt activity, induces skeletal muscle hypertrophy and increases exercise capacity in a mouse model of diet-induced obesity

**Figure 1:**
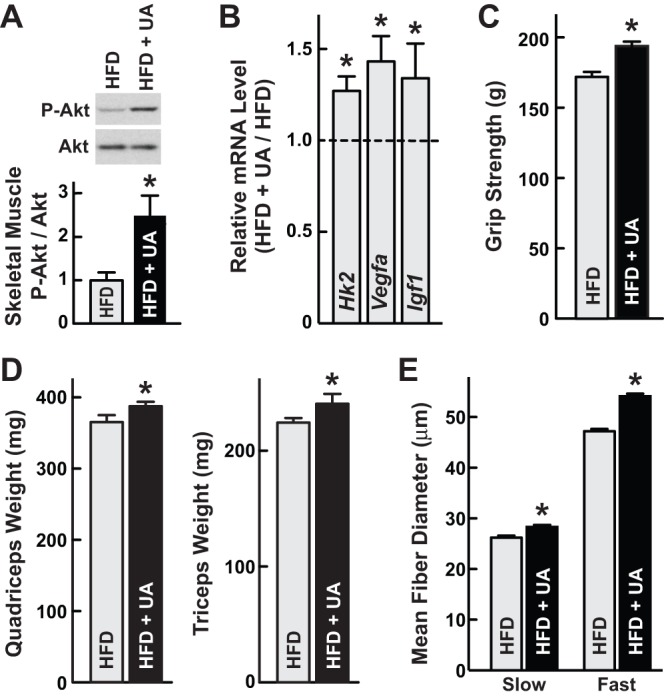
In mice fed a high fat diet, ursolic acid increases skeletal muscle Akt signaling, anabolic mRNA expression, grip strength, skeletal muscle mass, and fast and slow skeletal muscle fiber size. Mice were provided ad libitum access to high fat diet (HFD) lacking or containing 0.14% ursolic acid (UA) for 6 weeks. Data are means ± SEM. *P<0.05 by t-test. (A) Triceps muscles here harvested and subjected to SDS-PAGE and immunoblot analysis with anti-phospho(Ser473)-Akt and anti-Akt antibodies. *Upper*: representative immunoblots. *Lower*: Phospho-Akt (P-Akt) and total Akt levels were quantitated with densitometry. In each mouse, the phospho-Akt/total Akt ratio was normalized to the average phospho-Akt/total Akt ratio in mice fed HFD lacking UA. n = 5 mice per diet. (B) Quadriceps mRNA levels were determined using qualitative real-time RT-PCR (qPCR). Levels in UA-treated mice were normalized to the average levels in mice fed HFD lacking ursolic acid, which were set at 1. n = 10 mice per diet. (C) Grip strength. n = 10 mice per diet. (D) Weights of bilateral quadriceps and triceps brachii (triceps). n≥12 mice per diet. (E) Slow and fast muscle fiber diameters. Sections of triceps muscle were subjected to immunohistochemical analysis with anti-slow myosin and anti-fast myosin antibodies, and then fiber diameter was measured. Slow fibers: n≥50 fibers/triceps from 5 mice per condition. Fast fibers: n≥100 fibers/triceps from 5 triceps per condition.

To investigate the effects of ursolic acid in diet-induced obese mice, we provided 8-week-old male C57BL/6 mice ad libitum access to a high fat diet or a high fat diet supplemented with 0.14% ursolic acid for 6 weeks. This high fat diet (55% calories from fat) is known to cause obesity, as well as glucose intolerance and fatty liver disease [16], [17]. After 6 weeks on these diets, we harvested triceps muscle and examined steady-state Akt phosphorylation, a marker of Akt activity [18]. We found that ursolic acid increased Akt phosphorylation more than two-fold (Fig. 1A).

As an additional test of Akt activity, we measured levels of *hexokinase-II* (*Hk2*) and *vascular endothelial growth factor-A* (*Vegfa*) mRNAs; these transcripts are induced by Akt signaling in skeletal muscle and encode proteins that promote glucose utilization and blood vessel recruitment, respectively [8], [10], [19]. We found that ursolic acid increased both *Hk2* and *Vegfa* mRNAs (Fig. 1B). In our previous study of non-obese mice, we found that ursolic acid also increased the level of *Igf1* mRNA in skeletal muscle [5]. Local *Igf1* expression is an autocrine/paracrine mechanism that increases skeletal muscle IGF-I/Akt signaling and thus muscle growth [20]–[22]. We therefore examined ursolic acid's effects on skeletal muscle *Igf1* mRNA in high fat-fed mice, and found that ursolic acid increased it (Fig. 1B). These data provided further evidence that ursolic acid stimulates skeletal muscle Akt activity.

Since Akt signaling promotes skeletal muscle hypertrophy [7]–[13], we measured grip strength, muscle weight and muscle fiber size. We found that ursolic acid increased grip strength and skeletal muscle weight (Figs. 1C&D). We saw similar effects with 0.14% ursolic acid (Figs. 1C&D) and 0.27% ursolic acid (Fig. S1). Ursolic acid also increased the size of both fast and slow skeletal muscle fibers (Fig. 1E) without altering the ratio of fast to slow fibers. Thus, consistent with increased skeletal muscle Akt activity, ursolic acid induced skeletal muscle hypertrophy.

**Figure 2:**
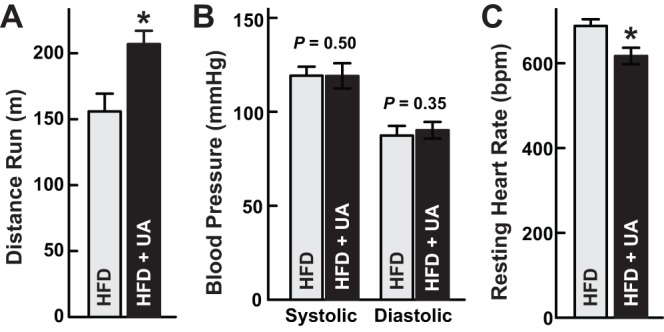
Ursolic acid increases exercise capacity, does not alter blood pressure, and reduces resting heart rate in high fat-fed mice. Mice were fed high fat diet (HFD) lacking or containing 0.27% ursolic acid (UA) for 17 weeks, and then exercise treadmill capacity was determined according to an established protocol [36] (A) and resting blood pressure and heart rate were determined with tail cuff plethysmography (B and C). Data are means ± SEM from ≥7 mice per diet. P-values were determined with t-tests. *P<0.05.

Since ursolic acid increased the size of both slow (oxidative) and fast (glycolytic) muscle fibers, we asked whether it might improve exercise capacity. To test this, we measured maximal running distance on an exercise treadmill. We found that ursolic acid-treated mice ran significantly farther than control mice (Fig. 2A). Treadmill running also reflects cardiovascular function, which we further evaluated by measuring resting blood pressure and heart rate. Ursolic acid did not alter blood pressure (Fig. 2B), and it induced a slight but significant reduction in resting heart rate (Fig. 2C). Thus, in addition to stimulating skeletal muscle Akt activity and muscle hypertrophy, ursolic acid improved exercise capacity, lowered resting heart rate and did not alter blood pressure.

### Ursolic acid reduces diet-induced obesity, glucose intolerance and fatty liver disease

**Figure 3:**
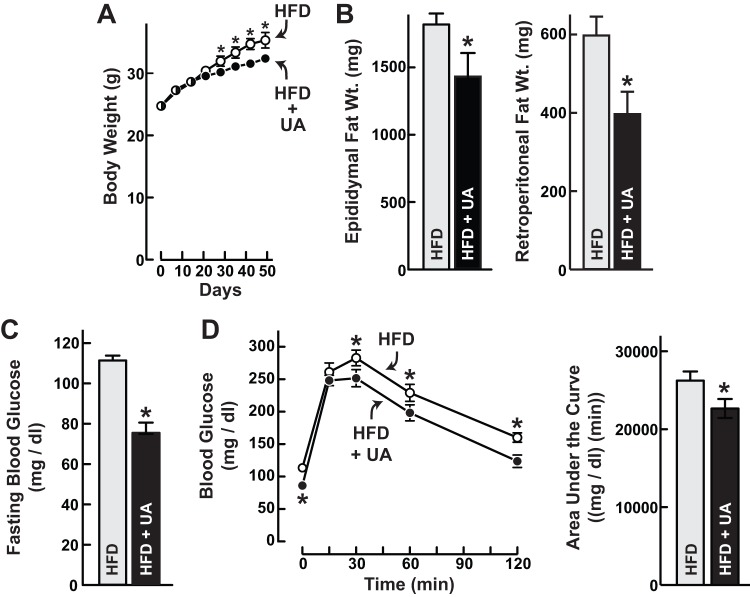
Ursolic acid reduces diet-induced obesity and glucose intolerance. Mice were provided ad libitum access to high fat diet (HFD) lacking or containing 0.14% ursolic acid (UA) for 6 weeks. Data are means ± SEM. *P<0.05 by t-test. (A) Total body weight was measured at the indicated times. n≥12 mice per diet. (B) Weights of bilateral epididymal and retroperitoneal fat pads. n = 10 mice per diet. (C) Fasting blood glucose levels. Mice were fasted for 16 h prior to tail vein glucose measurements. n≥12 mice per diet. (D) Glucose tolerance tests. Following a 16 h fast, 1 g/kg glucose was administered by i.p. injection at time  = 0 min. Blood glucose was then measured via the tail vein at the indicated times. n = 10 mice per diet. Left, blood glucose values. Right, areas under the curves.

Previous studies showed that skeletal muscle Akt activation [8] and ursolic acid [14], [15] reduce diet-induced obesity, white adipose tissue, glucose intolerance and fatty liver disease. Similarly, we found that mice that consumed high fat diet containing ursolic acid gained less weight than mice that consumed high fat diet without ursolic acid (Fig. 3A; Fig. S1). In addition, ursolic acid-treated mice had smaller epididymal and retroperitoneal fat pads (Fig. 3B; Fig. S1). These data indicated that ursolic acid reduced obesity. To evaluate glucose homeostasis, we measured fasting blood glucose. In the absence of ursolic acid treatment, fasting blood glucose was elevated at 109 ± 2 mg/dl (Fig. 3C). However, fasting blood glucose was normal (74 ± 5 mg/dl) in ursolic acid-treated mice (Fig. 3C). In addition to preventing fasting hyperglycemia, ursolic acid reduced glycemic excursion during a glucose tolerance test (Fig. 3D; Fig. S1). Thus, ursolic acid reduced both obesity and glucose intolerance.

**Figure 4:**
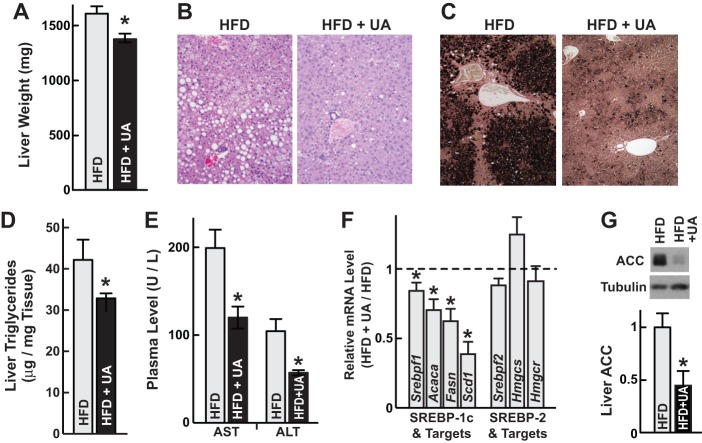
Ursolic acid reduces diet-induced fatty liver disease. Mice were provided ad libitum access to high fat diet (HFD) lacking or containing ursolic acid (UA) for 6 weeks. UA concentrations were 0.27% (B–C and E) or 0.14% (A, D and F–G). Data are means ± SEM. *P<0.05 by t-test. (A) Liver weights. n≥12 mice per diet. (B) Liver H&E-stained sections. 20x magnification. (C) Liver osmium-stained sections, 10x magnification. (D) Hepatic triglyceride content. n = 5 mice per diet. (E) Plasma aspartate aminotransferase (AST) and alanine aminotransferase (ALT) levels. n = 5 mice per diet. (F) Liver mRNA levels were determined using qPCR. Levels in UA-treated mice were normalized to the average levels in mice fed HFD lacking ursolic acid, which were set at 1. n = 10 mice per diet. (G) Livers were harvested and subjected to SDS-PAGE and immunoblot analysis with anti-ACC and anti-tubulin antibodies. Upper: representative immunoblots. Lower: ACC and tubulin levels were quantitated with densitometry. In each mouse, the ACC/tubulin ratio was normalized to the average ACC/tubulin ratio in mice fed HFD lacking ursolic acid. n = 6 mice per diet.

In addition to glucose intolerance, fatty liver disease is an important complication of diet-induced obesity [23]. Ursolic acid reduced liver weight (Fig. 4A; Fig. S1), hepatocellular steatosis (Figs. 4B and 4C) and hepatic triglyceride content (Fig. 4D); these data indicated reduced fatty liver disease. Ursolic acid also reduced plasma aminotransferases (Fig. 4E), suggesting reduced hepatocyte injury. At the molecular level, ursolic acid reduced the steady-state level of *Srebpf1* mRNA (Fig. 4F), which encodes SREBP-1c, a transcription factor that promotes lipogenesis and fatty liver disease [24], [25]. Accordingly, ursolic acid reduced expression of three key SREBP-1 target genes (*acetyl-CoA carboxylase 1* (*Acaca*), *fatty acid synthase* (*Fasn*) and *stearoyl Co-A desaturase-1* (*Scd1*)) (Fig. 4F), and it markedly reduced the level of acetyl-CoA carboxylase 1 (ACC) protein (Fig. 4G). In contrast, ursolic acid did not alter the levels of mRNA encoding SREBP-2 (*Srebpf2*) or SREBP-2-dependent mRNAs involved in cholesterol synthesis (3-hydroxy-3-methylglutaryl-CoA synthase-1 (*Hmgcs1*) and 3-hydroxy-3-methylglutaryl-CoA reductase (*Hmgcr*) (Fig. 4F). Thus, ursolic acid reduced obesity, glucose intolerance and fatty liver disease. All of these effects were consistent with increased skeletal muscle Akt activity [8].

### Ursolic acid increases brown fat

**Figure 5:**
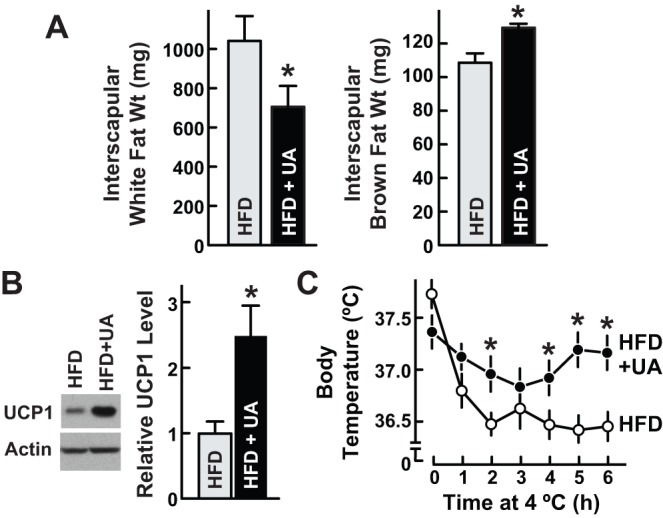
Ursolic acid increases interscapular brown fat. Mice were provided ad libitum access to high fat diet (HFD) lacking or containing 0.14% ursolic acid (UA) for 6 weeks. Data are means ± SEM. *P<0.05 by t-test. (A) Interscapular fat pads were harvested and dissected into white fat and brown fat components, which were then weighed. n = 10 mice per diet. (B) Protein from the entire interscapular fat pad was isolated and subjected to SDS-PAGE and immunoblot analysis with anti-UCP1 and anti-actin antibodies. *Upper*: representative immunoblots. *Lower*: UCP1 and actin levels were quantitated with densitometry. In each mouse, the UCP1/actin ratio was normalized to the average UCP1/actin ratio in mice fed HFD lacking UA, which was set at 1. n = 8 mice per diet. (C) Cold tolerance test. Following 6 weeks of HFD ± UA, a baseline rectal temperature was obtained at 21°C (t = 0 hours). Mice were then moved to 4°C, where rectal temperature was measured hourly. n = 8 mice per diet.

The interscapular fat pad contains two types of fat: white fat, which stores energy, and brown fat, which expends energy to generate heat and maintain body temperature. Importantly, brown fat shares developmental origins with skeletal muscle [26] and opposes the development of obesity [27]. Ursolic acid reduced interscapular white fat (Fig. 5A; Fig. S1), consistent with its effects on epididymal and retroperitoneal white fat (Fig. 3B). In contrast, ursolic acid increased interscapular brown fat (Fig. 5A). As an additional test, we measured expression of uncoupling protein 1 (UCP1), a brown fat marker, and found that ursolic acid increased UCP1 (Fig. 5B). Since brown fat is thermogenic, we hypothesized that ursolic acid-treated mice might be resistant to hypothermia. Indeed, ursolic acid reduced the decline of core body temperature at 4°C (Fig. 5C). Thus, in addition to increasing skeletal muscle, ursolic acid also increased brown fat.

### Ursolic acid increases energy expenditure

Since skeletal muscle and brown fat have relatively high rates of energy expenditure [28], we hypothesized that ursolic acid might increase energy expenditure. To test this, we used a system that also permitted simultaneous measurements of food intake and spontaneous activity. We studied mice that had consumed high fat food lacking or containing ursolic acid for either 3 days or 6 weeks. By testing both acute (3 days) and chronic (6 weeks) treatments, we could examine ursolic acid's effects before and after it altered body composition.

**Figure 6:**
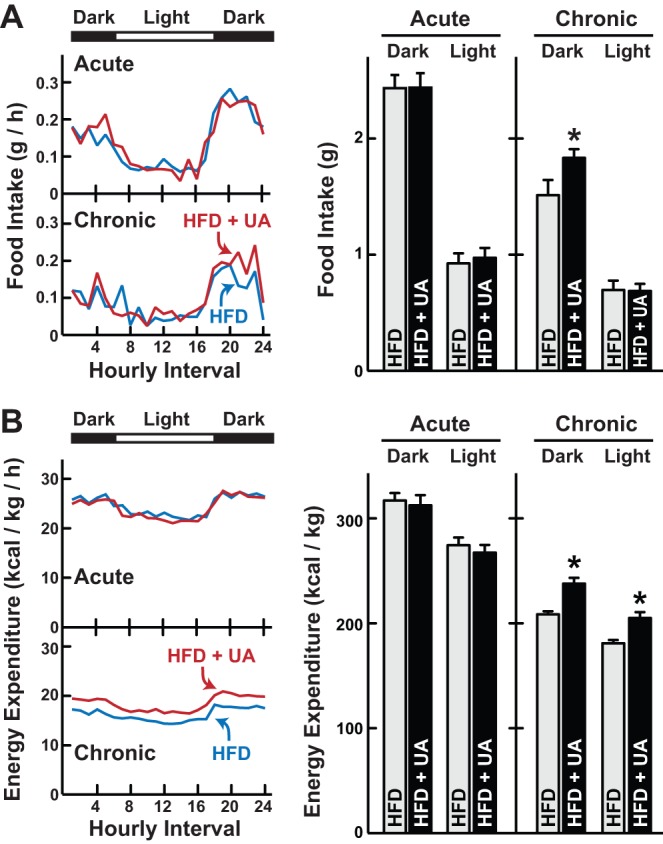
Chronic, but not acute, ursolic acid treatment increases food intake and energy expenditure. Mice were fed high fat diet (HFD) lacking or containing 0.27% ursolic acid (UA) for either 3 days (acute treatment) or 6 weeks (chronic treatment), and then food intake (A) and energy expenditure (B) were determined using a comprehensive lab animal monitoring system (CLAMS). *Left panels*: hourly measurements. Data are means from 12 mice per diet. *Right panels*: cumulative measurements during the dark and light periods. Data are means ± SEM from 12 mice per diet. *P-*values were determined with unpaired t-tests. *P<0.05.

One potential mechanism of obesity resistance is reduced food intake. However, acute treatment with ursolic acid did not alter food intake (Fig. 6A). Moreover, chronic treatment with ursolic acid increased food intake during the dark period, when mice are more active (Fig. 6A). These data ruled out reduced food intake as the mechanism of obesity reduction. In addition, increased food intake in the setting of obesity resistance was consistent with increased energy expenditure.

Fig. 6B shows the effects of acute and chronic ursolic acid treatment on energy expenditure. Acute treatment with ursolic acid had no effect (Fig. 6B). However, chronic treatment with ursolic acid significantly increased energy expenditure during both dark and light cycles (Fig. 6B) without significantly altering spontaneous activity (Fig. S2). These data indicated that ursolic acid reduced obesity by increasing resting energy expenditure. Furthermore, increased energy expenditure required chronic treatment with ursolic acid, which could be consistent with a requirement for skeletal muscle and brown fat growth.

## Discussion

Ursolic acid is contained in many edible fruits and herbs [3], [4]. We previously found that ursolic acid enhances skeletal muscle insulin/IGF-I signaling, leading to Akt activation, muscle hypertrophy, and reduced adiposity and blood glucose [5]. In the current study, we investigated ursolic acid's effects in the setting of a high fat diet, where muscle Akt activity is known to increase energy expenditure and reduce obesity and its complications [8]. We found that ursolic acid increased muscle Akt activity in high fat-fed mice. Moreover, as predicted by previous studies of transgenic mice expressing a constitutively active Akt construct specifically in skeletal muscle [8], the ursolic acid-mediated increase in skeletal muscle Akt activity was associated with skeletal muscle hypertrophy, increased energy expenditure, and reduced total body weight, white fat, glucose intolerance and hepatic steatosis.

Interestingly, we found that ursolic acid also increased brown fat, whose high rate of energy expenditure provides protection against obesity [27]. Although ursolic acid's effect on skeletal muscle is potentially sufficient to explain ursolic acid's effects on energy expenditure, thermogenesis, white fat, liver and glucose homeostasis [8], [29], we speculate that increased brown fat may also play an important role. We suggest a general model in which ursolic acid increases skeletal muscle and brown fat, leading to increased energy expenditure, and thus resistance to diet-induced obesity, fatty liver disease and glucose intolerance. This model provides a framework for further studies, including FDG-PET-CT-imaging to determine if the brown fat in ursolic acid-treated mice is metabolically active, and studies to determine whether ursolic acid affects brown fat, white fat and liver by direct or indirect mechanisms.

How ursolic acid increases brown fat remains uncertain. One possibility is that increased brown fat is a secondary effect of reduced insulating white fat. Another possibility is that ursolic acid increases sympathetic activity, which is known to expand brown fat [30]. Our findings that ursolic acid slightly decreased the resting heart rate, and did not alter blood pressure seem to argue against a systemic increase in sympathetic activity. However, sympathetic outflow to brown fat can be dissociated from sympathetic outflow to other tissues [31], [32]. Thus, it will be important to directly measure whether ursolic acid alters sympathetic outflow to brown fat.

A third possibility is that ursolic acid directly acts on brown fat to stimulate its growth. Ursolic acid increases skeletal muscle at least in part by enhancing ligand-dependent autophosphorylation of the IGF-I and insulin receptors [5]. Since brown fat and skeletal muscle arise from the same precursor cells [26], and since insulin/IGF-I signaling promotes brown fat growth [33], it is tempting to speculate that ursolic acid increases brown fat and skeletal muscle through a common molecular mechanism. This mechanism could involve multiple receptors based on a recent proteomic study showing that a closely related oleanane triterpenoid bound >500 proteins in HEK293 cells, including proteins involved in insulin/IGF-I signaling, AMP kinase signaling, JAK/Stat signaling, NFκB signaling and retinoic acid signaling [34]. It was therefore suggested that oleanane triterpenoids are multifunctional small molecules with many low affinity but biologically relevant targets [34]. Perhaps the same is true for other bioactive triterpenoids, such as ursolic acid. This is a challenging but important area for future investigation.

Other important areas for future investigation include studies to determine whether ursolic acid's effects on skeletal muscle mRNA expression (e.g. increased *Hk2*, *Vegfa* and *Igf1*) represent a reversal of changes that are induced by high fat feeding. It will also be interesting to determine whether ursolic acid might limit the induction of mRNAs that are typically induced in fatty liver (such as *Cd36*, *Mogat1* and *Pparg*), and whether higher or lower doses of ursolic acid might have differential effects on skeletal muscle, brown fat, white fat and liver.

In summary, we found that ursolic acid increases skeletal muscle, brown fat and energy expenditure in a mouse model of diet-induced obesity. These effects are associated with increased strength and exercise capacity, and reduced obesity, improved glucose tolerance and decreased hepatic steatosis. These pre-clinical data recommend further studies in humans. If ursolic acid has similar effects in mice and humans, then ursolic acid and/or structural analogs might be useful therapeutic agents for a number of increasingly common metabolic disorders, including skeletal muscle atrophy, obesity, type 2 diabetes and non-alcoholic fatty liver disease.

## Materials and Methods

### Ethics Statement

All experiments conformed to the National Institutes of Health Guide for the Care and Use of Laboratory Animals and were approved by the University of Iowa Institutional Animal Care and Use Committee. This protocol was approved under application number 1002026.

### Animals and diets

All studies utilized 6–8 week old male C57BL/6 mice, which were obtained from NCI and used for experiments within 3 weeks of their arrival. Prior to experiments, mice were maintained on standard chow (Harlan Teklad formula 7013). The high fat diet (HFD) was Harlan Teklad formula TD93075 and contained 55% of calories from fat. Ursolic acid was obtained from Enzo Life Sciences, and then sent to Harlan Teklad for incorporation into HFD. Mice were housed at 21°C except during the cold tolerance test, and 12 h light/12 h dark cycles were maintained throughout the study. Mice were colony caged except during CLAMS monitoring and for 2 days prior to and during the cold tolerance test, when mice were individually housed. Altogether, we performed seven independent experiments to test and confirm the effects of either 0.14% or 0.27% ursolic acid. Sample sizes for each experiment are described in the corresponding figure legends.

### Immunoblot analysis

Tissue samples were snap frozen in liquid N_2_, and then Triton-X 100 soluble protein extracts were prepared as previously described [5]. An aliquot of each extract was mixed with 0.2 volumes of sample buffer (250 mM Tris-HCl, pH 6.8, 10% SDS, 25% glycerol, 0.2% (w/v) bromophenol blue, and 5% (w/v) 2-mercaptoethanol) and heated for 5 min at 95°C, whereas a separate aliquot was used to determine protein concentration by the BCA kit (Pierce). Samples (25 μg) were subjected to SDS-PAGE, then transferred to Hybond-C extra nitrocellulose filters (Millipore). Immunoblots were performed at 4°C for 16 h using a 1∶2000 dilution of antibodies detecting total Akt, phospho-Akt (Ser473), ACC, or α-tubulin (all from Cell Signaling); UCP1 (Abcam); or actin (Santa Cruz).

### Quantitative Real-Time RT-PCR

Tissue samples were immediately placed in RNAlater (Ambion) and stored at −80°C until further use. Total RNA was extracted using TRIzol solution (Invitrogen). TRIzol-extracted mRNA was treated with DNase I using the Turbo DNA-free kit (Ambion). qPCR analysis of *Igf1*, *Hk2*, *Vegfa*, *Srebpf1, Acaca, Fasn, Scd1, Srebpf2, Hmgcs1* and *Hmgcr* was performed using TaqMan Gene Expression Assays (Applied Biosystems). First strand cDNA was synthesized from 2 μg of RNA using the High Capacity cDNA Reverse Transcription Kit (part no. 4368814; Applied Biosystems). The real time PCR contained, in a final volume of 20 μl, 20 ng of reverse transcribed RNA, 1 μl of 20X TaqMan Gene Expression Assay, and 10 μl of TaqMan Fast Universal PCR Master Mix (part no. 4352042; Applied Biosystems). qPCR was carried out using a 7500 Fast Real-Time PCR System (Applied Biosystems) in 9600 emulation mode. All qPCR reactions were performed in triplicate and the cycle threshold (Ct) values were averaged to give the final results. To analyze the data, we used the ΔCt method, with level of *36B4* mRNA serving as the invariant control.

### Grip strength measurements

Forelimb grip strength was determined using a grip strength meter equipped with a triangular pull bar (Columbus Instruments). Each mouse was subjected to 5 consecutive tests to obtain the peak value.

### Skeletal muscle fiber analysis

Triceps muscles were fixed in 10% neutral buffered formalin, embedded in paraffin, and then sectioned with a Microm HM355S motorized microtome (Microm, Walldorf, Germany). Slides were treated with Proteinase K for 5 min (fast myosin) or 10 min (slow myosin). Slides were blocked with 10% goat serum for 60 min, then treated with a 1∶500 dilution of primary antibody (Sigma-Aldrich #M8421 (slow myosin) or Sigma-Aldrich #M4276 (fast myosin)) in Dako diluent (Dako, #S0809). Slides were next treated with Affinpure Fab Fragment Goat anti-Mouse IgG (Jackson ImmunoResearch #115-007-003) diluted 1∶500 in 10% goat serum for 30 min. Following this, slides were treated with Biotin SP Fab Fragment Goat Anti-Mouse (Jackson ImmunoResearch #115-066-072) diluted 1∶500 in 10% goat serum for 30 min. Signal was developed with DAB (Vector, SK-4100) for 5 min at 22°C, then slides were rinsed once with 1x wash buffer before adding DAB Enhancer (Vector, SK-4100) for 3 min. Slides were then counterstained with hematoxylin (Surgipath) and after air-drying for 20 min, coverslips were mounted with Vectashield (Vector, #H-1400) before visualization with an Olympus IX-71 microscope equipped with a DP-70 camera. Image analysis was performed using ImageJ software, and myofiber diameter was measured using the lesser diameter method, as described elsewhere [35].

### Exercise treadmill testing

Mice were acclimated to a motor-driven open treadmill set at 14 M/min at 0% grade and a shock grid set at 0.2 mA (Columbus Instruments model #1055MSD) for 5 min per day for 2 days. On the following day, exercise tolerance was determined at a constant treadmill incline of 10% as described previously [36]. For the first 5 min, the treadmill was set 10 M/min. We then increased the speed by 2 M/min every 2 min. The test was terminated when mice contacted the shock grid for 10 seconds.

### Heart rate and blood pressure

Mice were acclimated to tail cuff plethysmography (BP-2000, Visitech Systems) 3 times weekly for 2 weeks before heart rate and blood pressure measurements were obtained.

### Glucose tolerance tests

Mice were acclimated to human handling weekly for 6 weeks, then fasted for 16 h (from 1700 to 0900 h). Blood glucose was then obtained from the tail vein with an Accucheck Aviva glucose meter. Mice then received an i.p. injection of glucose (1 g/kg body weight) and blood glucose was measured 15, 30, 60 and 120 min later.

### Liver histology, lipid analysis and plasma aminotransferase measurements

Liver tissue was fixed in 10% neutral buffered formalin, embedded in paraffin, sectioned with a Microm HM355S motorized microtome (Microm, Walldorf, Germany), and then stained with hematoxylin and eosin or with osmium. For osmium staining, sections were incubated in 2% (w/v) osmium tetroxide and 5% (w/v) potassium dichromate for 7 hours at 22°C, followed by a 2 hour tap water rinse. Osmium stained tissues were routinely processed, paraffin embedded, sectioned and stained with neutral red. Sections were examined on an Olympus IX-71 microscope equipped with a DP-70 camera. For lipid analysis, liver samples were snap frozen in liquid N_2_, and then shipped to Vanderbilt Mouse Metabolic Phenotyping Core, where lipid levels were measured as described previously [37], [38]. Mouse plasma AST and ALT were measured using the VITROS 350 Chemistry System.

### Cold tolerance tests

Mice were acclimated to a rectal temperature probe (Oakton Instruments) every other day for one week, and then fed HFD lacking or containing 0.14% ursolic acid for 6 weeks. Mice were then moved to individual cages for two days before starting the test. On the day of the test, a baseline rectal temperature measurement was obtained at 21°C. Mice were then transferred for 6 hours to a 4°C room, where rectal temperature was measured hourly.

### Analysis of energy expenditure, food intake and activity

Mice were moved to comprehensive lab animal monitoring systems (CLAMS; Columbus Instruments) at 1800 h and acclimated to the CLAMS for 54 h. We then obtained food intake, indirect calorimetry, and X- and Y-axis activity measurements over the next 24 h. Activity was defined as ≥2 beam-breaks in one axis within a 30 sec period. The light/dark cycle and temperature were constant before and during CLAMS.

## Supporting Information

Figure S1 **In high fat-fed mice, 0.27% ursolic acid increases skeletal muscle and brown fat while reducing obesity, glucose intolerance and fatty liver disease.** Mice were provided ad libitum access to high fat diet (HFD) lacking or containing 0.27% ursolic acid (UA) for 6 weeks. Data are means ± SEM from ≥7 mice per diet. *P<0.05 by t-test. (A) Grip strength. (B) Weights of bilateral quadriceps and triceps. (C) Total body weight. (D) Weight of bilateral epididymal and retroperitoneal fat pads. (E) Fasting blood glucose. (F) Intraperitoneal glucose tolerance tests. (G) Liver weight. (H) Weight of bilateral interscapular white and brown fat components.
(EPS)

Figure S2 **Ursolic acid treatment does not alter spontaneous activity.** Mice were fed high fat diet (HFD) lacking or containing 0.27% ursolic acid (UA) for either 3 days (acute treatment) or 6 weeks (chronic treatment), and then spontaneous activity in the X- and Y-axes was determined using a comprehensive lab animal monitoring system (CLAMS). Data are means ± SEM from 12 mice per diet. *P*-values were determined with unpaired t-tests. *P<0.05. *Left*: hourly activity measurements. *Right*: cumulative activity measurements during the dark and light periods. In these experiments, we also measured food intake and energy expenditure; those data are shown in Fig. 6.
(EPS)
